# Myopathy-Sensitive G-Actin Segment 227-235 Is Involved in Salt-Induced Stabilization of Contacts within the Actin Filament

**DOI:** 10.3390/ijms22052327

**Published:** 2021-02-26

**Authors:** Joanna Gruszczynska-Biegala, Andrzej Stefan, Andrzej A. Kasprzak, Piotr Dobryszycki, Sofia Khaitlina, Hanna Strzelecka-Gołaszewska

**Affiliations:** 1 Department of Muscle Biochemistry, Nencki Institute of Experimental Biology, 02-093 Warsaw, Poland; jgruszczynska@imdik.pan.pl (J.G.-B.); a.stefan@nencki.gov.pl (A.S.); a.kasprzak@nencki.gov.pl (A.A.K.); h.strzelecka@nencki.gov.pl (H.S.-G.); 2 Molecular Biology Unit, Mossakowski Medical Research Institute Polish Academy of Sciences, 02-106 Warsaw, Poland; 3 Faculty of Chemistry, Wrocław University of Technology, 50-370 Wroclaw, Poland; piotr.dobryszycki@pwr.edu.pl; 4 Laboratory of Cytology of Unicellular Organisms, Institute of Cytology, Russian Academy of Sciences, 194064 St. Petersburg, Russia

**Keywords:** actin filaments, actin proteolysis, subtilisin, salt-induced stabilization, C-terminus, Segment 227-235, actin-associated myopathy

## Abstract

Formation of stable actin filaments, critically important for actin functions, is determined by the ionic strength of the solution. However, not much is known about the elements of the actin fold involved in ionic-strength-dependent filament stabilization. In this work, F-actin was destabilized by Cu^2+^ binding to Cys374, and the effects of solvent conditions on the dynamic properties of F-actin were correlated with the involvement of Segment 227-235 in filament stabilization. The results of our work show that the presence of Mg^2+^ at the high-affinity cation binding site of Cu-modified actin polymerized with MgCl_2_ strongly enhances the rate of filament subunit exchange and promotes the filament instability. In the presence of 0.1 M KCl, the filament subunit exchange was 2–3-fold lower than that in the MgCl_2_-polymerized F-actin. This effect correlates with the reduced accessibility of the D-loop and Segment 227-235 on opposite filament strands, consistent with an ionic-strength-dependent conformational change that modulates involvement of Segment 227-235 in stabilization of the intermonomer interface. KCl may restrict the mobility of the α-helix encompassing part of Segment 227-235 and/or be bound to Asp236 at the boundary of Segment 227-235. These results provide experimental evidence for the involvement of Segment 227-235 in salt-induced stabilization of contacts within the actin filament and suggest that they can be weakened by mutations characteristic of actin-associated myopathies.

## 1. Introduction

Actin filament is stabilized by intersubunit interactions along the two long-pitch helical strands and by lateral contacts between the strands. The atomic models of the actin filament derived by fitting the atomic structures of G-actin to X-ray diffraction patterns of oriented gels of F-actin [1,2,3,4] and from cryo-electron microscopy data [5,6,7] predict that D-loop (Residues 39–52) on the top of actin Subdomain 2 is extended towards a hydrophobic groove between Subdomains 1 and 3 of the longitudinally adjacent protomer,^,^ including a stretch of Residues 139–143 [1,3,5,6] and a cluster of the C-terminal residues [1,3]. Although involvement of the C-terminus in the inter-protomer contacts was not visualized by cryo-electron microscopy [5,6,7], several lines of experimental evidence suggest that the C-terminus of actin participates in the longitudinal contact with Residues 41–45 of D-loop as predicted by the earlier models. In addition to the quenching of the fluorescence of dansyl ethylenediamine (DED) attached to Gln41 by copper ion (Cu^2+^) binding to Cys374 in F-actin but not in G-actin [8], this evidence includes intrastrand cross-linking of Cys374 to Gln41 [9], cross-linking and formation of an interprotomer disulfide bond between Cys374 and Cys41 substituted for Gln41 in yeast mutant F-actin [10,11,12], and intermolecular stacking of pyrene probes attached to Cys374 and Cys41 in copolymers of pyrene labeled wild type and Q41C/C374S mutant actin [13]. These data imply that the inter-protomer interaction between the D-loop and the C-terminus is either transient or structurally flexible [7]. In line with this suggestion, the cryo-electron microscopy structure of phalloidin-bound F-actin revealed conformational changes in the D-loop causing it to extend and establish contacts not only with residues Tyr148, Thr143 and Tyr169, but also with the C-terminal Phe375 of the neighboring actin [14]. The allosteric relationships between the D-loop and the C-terminus of actin molecule [15] can also be involved in these interactions. 

The interstrand contacts in F-actin models are also a point of discussion. In the “hydrophobic plug” hypothesis, Holmes et al. [1] proposed that the D-loop and C-terminus make contacts with Residues 262-274 (plug residues) in Subdomain 4 of an adjacent protomer from the opposing strand. This hypothesis gained support from intermolecular cross-linking and disulfide bond formation between these structural elements, and from fluorescence studies on copolymers of pyrene-labeled wild type yeast actin and its mutants with cysteine residues introduced either at position 41 or 265 and Cys374 replaced with alanine [10,13,16]. However, in the recent F-actin models, the hydrophobic plug contacts Residues 39–42 of D-loop along the filament strand [5] and Residues 173 [5,6,7] or 170–174 [3] across the strand but does make any prominent interaction with C-terminal residues within the protomers of the opposite strand. This interstrand interaction is formed by salt bridges rather than by hydrophobic interactions [5,7]. 

Cooperative interaction between the neighboring F-actin protomers has been also suggested to involve Residues 223-230, based on F-actin reconstructions from its electron microscopic images [17] and emerged from using slow normal modes of G-actin to refine the F-actin model against X-ray fiber diffraction data [18]. This possibility is supported by the intermolecular effects of proteolytic cleavage of D-loop at Gly42 on proteolytic susceptibility of Segment 227-235 [19] partially overlapping the 223-230 helix in Subdomain 4, by radiolytic footprinting data showing diminished solvent accessibility of Met227 in 50 mM KCl-polymerized magnesium (Mg)-F-actin as compared with Mg-actin oligomers produced by 0.2 mM MgCl_2_ [20] and by excimer formation between pyrene maleimids attached to Cys224 and Cys374 in fluorescence spectrum of yeast actin E224C mutants [21]. 

The apparent controversy of the structural and biochemical data available to date may be due to dynamical nature of actin filaments determined by conformational state of the flexible loops that form the intermonomer interface. In turn, conformation of the loops is affected by the kind of tightly bound nucleotide [22,23] and cation [24] as well as by polymerizing conditions [25,26]. Therefore, the aim of this work was to examine the effects of solvent conditions and Ca^2+^/Mg^2+^ replacement at the tightly bound cation site on the dynamic properties of F-actin, and to correlate these effects with involvement of Segment 227-235 in the contact formation. Destabilization of F-actin by Cu^2+^ binding to Cys374 [8] that is not directly involved in the intermonomer contacts [5,6,7] but makes them transient or structurally flexible [7,14] proved to be a convenient tool to reveal allosteric effects stabilizing the filament. Our results provide experimental evidence for involvement of Segment 227-235 in salt-induced stabilization of contacts within the actin filament and suggest that they can be weakened by the modifications produced in Segment 227-235 by mutations characteristic of myopathy [27,28].

## 2. Results

### 2.1. Effects of the Type of Cation Tightly Bound to Actin and of Solvent Conditions on ATP Hydrolysis in Steady-State F-Actin Solutions

**Figure 1:**
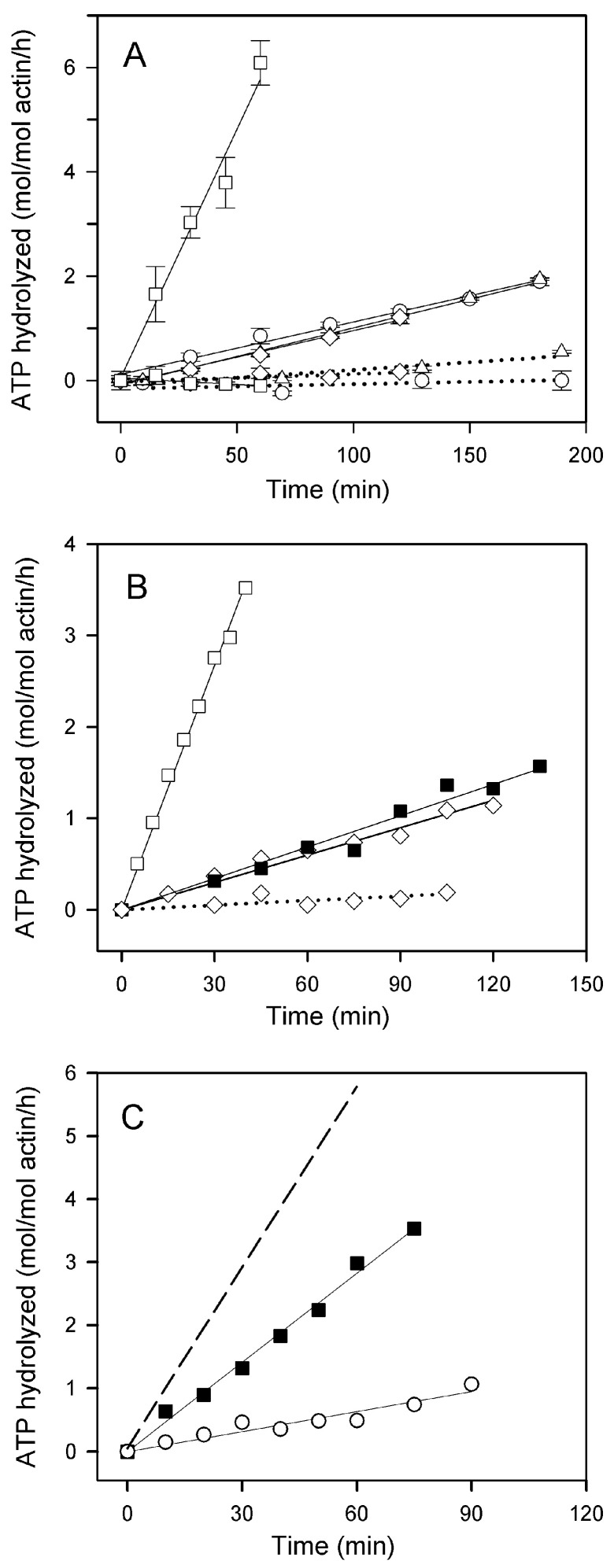
Effects of Cu^2+^ binding on steady-state ATP hydrolysis in F-actin solutions at various solvent conditions. (**A**) Ca-G-actin (◯,△) and Mg-G-actin (☐, ◇) (10 µM) were polymerized either with 2 mM CaCl_2_ (◯) or MgCl_2_ (☐), or with 0.1 M KCl (△, ◇). The F-actin solutions were incubated at 25 °C in the absence (dotted lines) or in the presence of 10 µM CuCl_2_ (solid lines). At time intervals, aliquots of the solutions were withdrawn for determination of P_i_ concentration as described in Materials and Methods. The concentration of P_i_ in samples taken 15 min after addition of CuCl_2_ (time zero in the figure) was subtracted from the values obtained for the longer incubation times. Mean values (±SD) from 3–5 independent experiments are presented. (**B**) ATP hydrolysis in steady-state solutions of Mg-F-actin (10 µM) polymerized with 2 mM MgCl_2_ (☐), 0.1 M KCl (◇), or 2 mM MgCl_2_/0.1 M KCl (◼) incubated at 25 °C in the absence (dotted line) or in the presence of 10 µM CuCl_2_ (solid lines). The liberation of P_i_ was measured as in (A). (**C**) Ca-G-actin (10 µM) was polymerized with 2 mM CaCl_2_ (◯) or with 2 mM MgCl_2_ (◼). The F-actin solutions were incubated at 25 °C in the presence of 10 µM CuCl_2_ and steady-state ATP hydrolysis was measured as in (A). The dashed line shows ATP hydrolysis by Mg-F-actin polymerized with MgCl_2_.

Hydrolysis of Adenosine triphosphate (ATP) in F-actin solutions at steady-state reflects the exchange of F-actin subunits with the monomer pool. It results from preferential dissociation of subunits carrying bound ADP from the slowly growing pointed end of the filament, exchange of the bound ADP for ATP on the monomers, and reincorporation of ATP-monomers into the filament followed by hydrolysis of their bound ATP (for a review see [29,30,31,32]). In agreement with earlier reports [33,34,35], the ATPase activity of fully polymerized actin, measured at 25 °C, was very low (below 0.2 mol of P_i_ liberated/mol of actin/h, Figure 1A, dotted lines) which precludes reliable evaluation of its dependence on the type of the bound cation or polymerizing salt. Striking effects of these factors were, however, observed with Cu^2+^-treated actins: this treatment increased the ATPase activity of Mg-G-actin polymerized with MgCl_2_ to 5–6 moles of ATP hydrolyzed/mol of actin/h (Figure 1A, solid lines), whereas the rates of ATP hydrolysis by both Ca-actin and Mg-actin polymerized with 0.1 M KCl, as well as by Ca-actin polymerized with CaCl_2_, were enhanced to only 0.60–0.65 moles/mol of actin/h. The stabilizing effect of increasing the ionic strength of the solution is also apparent from data in Figure 1B showing that the presence of 0.1 M KCl in addition to 2 mM MgCl_2_ reduced the steady-state ATPase activity of Mg-F-actin to the level observed with this actin polymerized with KCl alone. Similar lowering of the rate of steady-state ATP hydrolysis (measured at 37 °C) by addition of KCl, NaCl or LiCl to MgCl_2_-polymerized Ca-actin destabilized by glutathione modification of Cys374 has been reported previously [36].

In numerous studies on F-actin, Ca-G-actin polymerized with MgCl_2_ has been used. As shown in Figure 1C, the steady-state ATPase activity of Cu^2+^-treated Ca-G-actin polymerized with 2 mM MgCl_2_ (2.8 moles P_i_/mol of actin/h) was intermediate between those of Cu^2+^-treated Ca-G-actin polymerized with 2 mM CaCl_2_ (Ca-F-actin) and Mg-G-actin polymerized with 2 mM MgCl_2_ (Mg-F-actin). This is entirely consistent with only partial replacement of G-actin-bound Ca^2+^ by Mg^2+^ under these conditions [37,38], resulting in a copolymer of Ca- and Mg-actin.

### 2.2. Effects of the Type of Cation Tightly Bound to Actin and of Solvent Conditions on the Relative Filament Number

**Figure 2:**
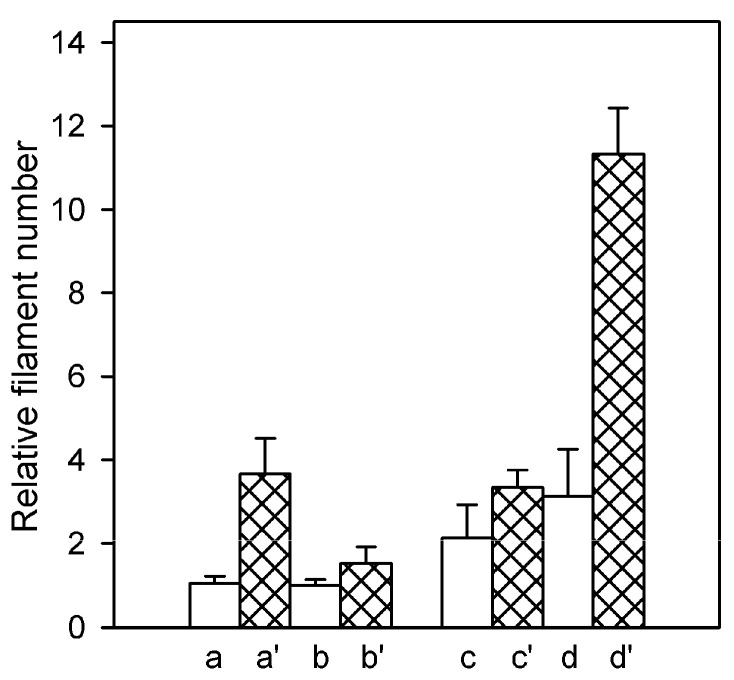
Cu^2+^-induced fragmentation of F-actin filaments. Ca-actin (a, b, and a’, b’) and Mg-actin (c, d, and c’, d’) were polymerized, at 25 °C, either with 2 mM CaCl_2_ (a, a’) or MgCl_2_ (d, d’), respectively, or with 0.1 M KCl (b, b’, c, c’). Relative number concentration of the filaments in 10 µM steady-state F-actin solutions was determined as described in Materials and Methods, before (a–d) and after a 15-min incubation with equimolar CuCl_2_ (a’–d’). Mean values (±SD) from 8–10 measurements on 2 independent actin preparations are presented.

The rate of the polymer subunit exchange underlying the steady-state ATPase activity of F-actin depends on the rate constants of the monomer dissociation from and association to the filament ends and on the number concentration of the filament ends. To get an insight into the nature of Cu^2+^-induced changes leading to the acceleration of the subunit exchange, we have determined relative number concentrations of filaments in the assayed F-actin solutions. This was done by comparing their abilities to nucleate actin polymerization when diluted into solutions of pyrene-labeled G-actin supplemented with 0.1 M KCl shortly before addition of the F-actin seeds. The initial rate of increase in the pyrene fluorescence, indicating elongation of the seeding filaments by addition of pyrene-labeled monomers to their ends, is proportional to the number concentration of the filaments [35]. As depicted in Figure 2, these measurements revealed higher filament number concentration in Mg-F-actin than in Ca-F-actin solutions: twofold in the presence of KCl and threefold in MgCl_2_ or CaCl_2_, respectively.

**Figure 3:**
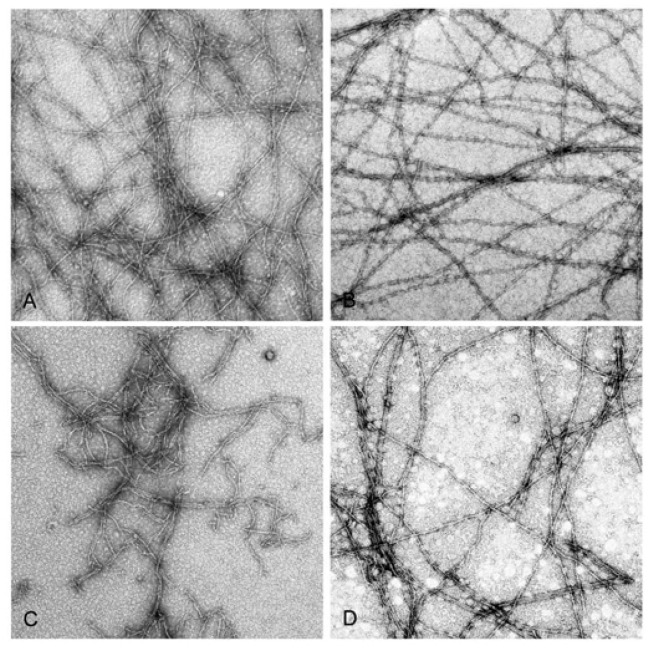
Electron micrographs of F-actin filaments before and after modification with CuCl_2_. Mg-G-actin (10 µM) was polymerized with 2 mM MgCl_2_ (**A**,**C**) or 0.1 M KCl (**B**,**D**) at 25 °C, and polymerization progress was monitored by measuring the increase in light scattering intensity. When the steady state of polymerization was reached, the solutions were negatively stained as described in Materials and Methods, before (**A**,**B**) and after a 15-min incubation with CuCl_2_ equimolar to actin (**C**,**D**). Pictures were taken at a magnification of 6000×.

This difference is in line with the higher rate of nuclei formation by Mg-G-actin (for review see [39,40], which naturally leads to larger number of the filaments at the expense of their lengths. Copper binding resulted in enhancement of the filament number concentration under all assayed conditions, indicating diminished mechanical stability of the modified filaments leading to their fragmentation. The enhancement was larger (about 3.5-fold) when the actins were polymerized with 2 mM CaCl_2_ or MgCl_2_ rather than in the presence of 0.1 M KCl (1.5-fold) independent of the type of tightly bound cation (Figure 2). These data show again a stabilizing effect of elevated ionic strength. Filament fragmentation upon Cu^2+^ binding and the protective effect of ionic-strength enhancement was confirmed by electron microscopic investigation of negatively stained preparations of Mg-actin polymerized with either 0.1 M KCl or 2 mM MgCl_2_ (Figure 3). 

**Figure 4:**
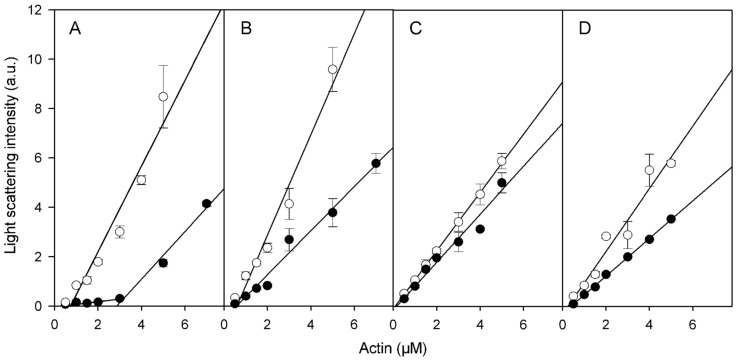
Effects of Cu^2+^ binding on the critical concentration for actin polymerization under various solvent conditions. Ca-G-actin (**A**,**B**) and Mg-G-actin (**C**,**D**) were polymerized either with 2 mM CaCl_2_ (**A**) or MgCl_2_ (**D**), or with 0.1 M KCl (**B**,**C**). Part of each F-actin solution was then incubated with CuCl_2_ equimolar to actin for 15 min. The solutions of non-modified (empty symbols) and Cu^2+^-modified F-actins (filled symbols) were diluted to the concentrations indicated and, after incubation at 25 °C for 2 h, the extent of polymerization was determined by measuring light scattering intensity at 450 nm and 90°. Mean values (±SD) from 3–5 independent experiments are presented.

In preparations of Cu^2+^-treated actin polymerized with MgCl_2_, numerous short filaments are seen (Figure 3C), whereas in similarly treated preparations of actin polymerized with KCl long filaments prevail (Figure 3D). In line with this, Cu^2+^ binding decreased the slopes of the curves showing dependence of light scattering of the corresponding F-actin solutions on actin concentration (Figure 4) which may indicate Cu^2+^-induced changes in the filament length and/or flexibility. It did not result, however, in any substantial change in critical G-actin concentration for polymerization (*C_c_*) except for CaCl_2_-polymerized Ca-actin for which *C_c_* increased from 0.7 μM to 2.8 µM (Figure 4), without any significant effect on the degree of this actin polymerization comparing with other Cu-modified actin species ((Figure 4). On the other hand, the high amount of G-actin in the Cu^2+^-treated CaCl_2_- polymerized Ca-F-actin may promote the filament instability which makes subunit exchange in this actin similar to that in the KCl-polymerized Mg-actin (Table 1).

When the rates of steady-state ATP hydrolysis in solutions of Cu^2+^-treated F-actins are normalized to the same number of the filament ends (Table 1) the difference between MgCl_2_-polymerized Mg-F-actin and other F-actin species diminishes to only three-fold or even less. This indicates that the particularly high ATPase activity of this F-actin results from both the tightly-bound cation- and polymerizing salt-dependent changes in the polymer structure that can promote filament breaking. Thereby, unexpectedly high level of subunit exchange in the Cu-modified KCl-polymerized Ca-F-actin suggests a Cu-induced destabilization of these actin filaments.

### 2.3. Probing Interprotomer Contacts by DED-F-Actin Fluorescence Measurements

**Figure 5:**
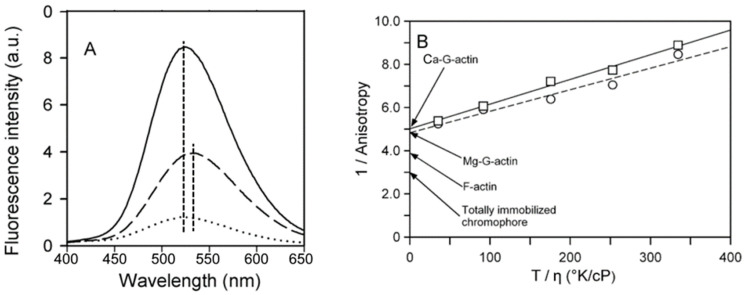
Effects of polymerization and Cu^2+^ binding on the fluorescence properties of DED-labeled actin. (**A**) Emission spectra of dansyl ethylenediamine (DED)-labeled actin. CaATP-G-actin (10 μM, 50% DED-labeled) was polymerized with 2 mM CaCl_2_. The fluorescence emission spectra of G-actin (dashed line) and F-actin (solid line) were recorded with excitation at 334 nm. Dotted line shows the spectrum of DED-F-actin after 15-min incubation with CuCl_2_ equimolar to actin. CuCl_2_ treatment of DED-G-actin did not produce any change in the spectrum. The vertical lines indicate the maxima of the fluorescence spectra. (**B**) Perrin plot for the fluorescence anisotropy of DED-labeled Ca- and Mg-G-actin. The anisotropy of 10 μM, 55% DED-labeled Ca-G-actin (☐) or Mg-G-actin (◯) was measured at 25 °C as a function of sucrose concentration (0–46%) in the presence of 10 mM Hepes, 0.5 mM ATP and 0.1 mM CaCl_2_ or 0.1 mM MgCl_2_, respectively. The excitation and emission wavelengths were 334 and 535 nm, respectively. The extrapolated fundamental anisotropies (A_0_^app^) of DED-labeled actins were obtained by extrapolating 1/A values to the infinite viscosity of the solution. The values were 0.20 ± 0.005, *n* = 4 for Ca-G-actin and 0.21 ± 0.01, *n* = 3 for Mg-G-actin, respectively; n corresponds to the number of independent measurements for each sucrose concentration. On the average, the SD values for A (anisotropy) for Ca-G-actin were 0.001 and 0.002 for Mg-G-actin. Using error propagation rules, the corresponding deviations for 1/A were computed to be in the range 0.1 to 0.2, too small to depict them on the graph. The anisotropies for F-actin polymerized by three salts were measured directly. For KCl-polymerized F-actin, A = 0.240 ± 0.004, *n* = 16; for Ca^2+^-polymerized F-actin A = 0.242 ± 0.004, *n* = 8; for Mg^2+^-polymerized F-actin A = 0.254 ± 0.002, *n* = 3. Since differences between the anisotropy values were small they collectively were marked as F-actin on the graph. The limiting anisotropy value (A_0_ = 0.335) for dansyl chromophore was taken from [42].

Dansyl-based probes such as DED are sensitive to follow changes in the environment of Gln41 within D-loop of actin during its polymerization. We have previously shown that when either CaATP- or MgATP-G-actin was polymerized with 0.1 M KCl, the quantum yield of the dye increased over twofold with a concomitant 10-nm blue shift in the fluorescence maximum [41] Similar effect of polymerization of Ca-ATP-G-actin with 2 mM CaCl_2_ is shown in Figure 5A. 

Steady-state anisotropy measurements on DED-labeled actin indicated no difference either between MgATP- and CaATP-G-actin (not shown) or between F-actins polymerized by KCl, CaCl_2_ or MgCl_2_ (A ≈ 0.245; Figure 5B). The anisotropies of DED-conjugated actin at infinite viscosity obtained from the Perrin plots (0.20 ± 0.005 and 0.21 ± 0.01 for Ca-G-actin and Mg-G-actin, respectively; Figure 5B) were found to be significantly lower than those for F-actin (see above and Figure 5B) or a totally immobilized chromophore (A_0_ = 0.335 [42]), which is in line with the structural data on high rotational mobility of D-loop [1,3,4,5,6]. However, even in F-actin, the label or the polypeptide chain was only partially immobilized. The value of the semi-angle of rotation θ calculated from Equation (2)was 31° for Ca-G-actin, 30° for Mg-G-actin and 25° for F-actin. A similar value (θ = 21°) was obtained for dansyl cadaverine conjugated with Gln-41 of F-actin, a label with a much longer spacer that allows for more orientational freedom [43]. This suggests that the mobility of both labels in F-actin originated from the protein segment to which the dyes were attached rather than from the rotation of the dyes alone. It has earlier been shown that Cu^2+^ binding to Cys374 of actin quenches the fluorescence of DED-labeled F-actin by more than 50% due to an intermolecular effect reflecting an exposure of D-loop to the solvent [8].

**Figure 6:**
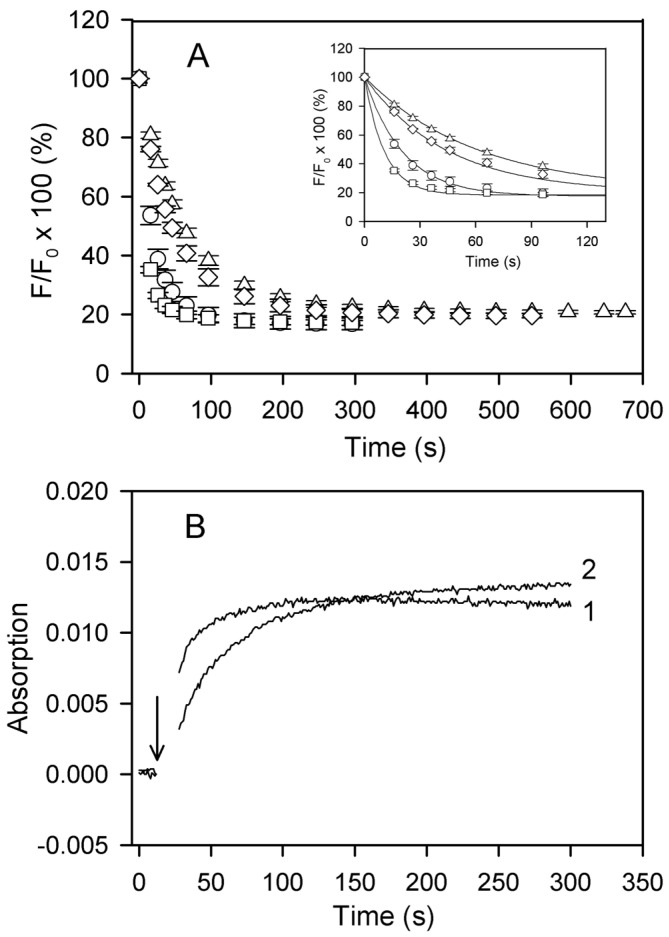
Time courses of Cu^2+^ binding to F-actin at various solvent conditions. (**A**) DED-labeled Ca-G-actin (◯,△) and Mg-G-actin (☐,◇) (10 µM, 50% DED-labeled) were polymerized either with 2 mM CaCl_2_ (◯) or MgCl_2_ (☐), or with 0.1 M KCl (△, ◇). At time zero, the F-actin solutions were supplemented with 10 µM CuCl_2_ and the fluorescence of DED was measured at 525 nm after excitation at 334 nm. Mean values (± SD) from 3–5 independent experiments are presented. A fit of the data to the first-order kinetic equation (inset) yielded the observed rate constants of 0.05 s^−1^ (◯), 0.09 s^−1^ (☐), 0.017 s^−1^ (◇), and 0.023 s^−1^(△). (**B**) Mg-G-actin (15 µM) was polymerized with either 2 mM MgCl_2_ or 0.1 M KCl in one of the compartments of a tandem quartz cuvette. The other compartment contained an equal volume of 15 µM CuCl_2_ in buffer Mg-G supplemented with 2 mM MgCl_2_ or 0.1 M KCl, respectively. Absorption at 348 nm was recorded before and after the solutions of the two compartments were mixed at the time indicated by arrow. Trace 1, actin polymerized with MgCl_2_; trace 2, actin polymerized with KCl.

As one can see in Figure 6A, the final extent of the fluorescence quenching is independent of the kind of divalent cation, Ca^2+^ or Mg^2+^, tightly bound to actin, or the type of polymerizing salt. However, the rate of the Cu^2+^-induced decrease in the fluorescence intensity of DED-labeled Mg-F-actin was about twofold higher than with Ca-F-actin when these actins were polymerized with 2 mM MgCl_2_ or CaCl_2_, respectively. This divalent cation-dependent kinetic difference was largely attenuated, and the fluorescence change was considerably slowed down when the actins were polymerized with 0.1 M KCl. The apparent rate constants are given in the legend to Figure 6A. 

As illustrated in Figure 6B, the kinetic differences were also observed when time courses of Cu^2+^ binding to actin were monitored by measuring absorption at 348 nm [31], which confirms that accessibility of the C-terminus is rate-limiting for the fluorescence quenching of DED-F-actin [8]. Similar final amplitudes but slower rates of the fluorescence quenching in Ca-F-actin rather than in Mg-F-actin and, in particular, in the presence of 0.1 M KCl as compared with 2 mM CaCl_2_ or MgCl_2_ imply tightly-bound Ca^2+^-dependent and ionic strength-dependent restriction of the accessibility of Cys374 in F-actin. The data in Figure 6 also show that these fluctuations are more sensitive to solvent conditions (ionic strength) than to the type of divalent cation tightly bound to actin. 

### 2.4. Probing Interprotomer Contacts in F-Actin by Limited Proteolysis with Subtilisin

**Figure 7:**
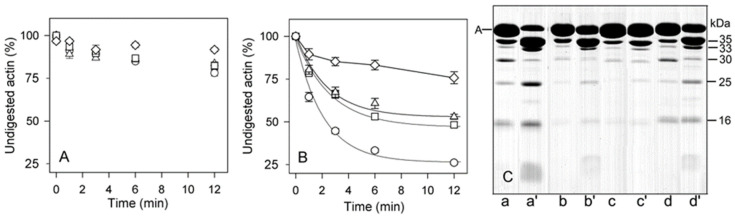
Effects of solvent conditions on subtilisin digestion of unmodified and Cu^2+^-modified F-actin. Ca-G-actin (◯, △) and Mg-G-actin (☐, ◇) (10 µM) were polymerized either with 2 mM CaCl_2_ (◯) or MgCl_2_ (☐), or with 0.1 M KCl (△, ◇). The F-actins were digested with subtilisin at an enzyme/protein mass ratio of 1:200 at 25 °C before (**A**) and after 15-min incubation with 10 µM CuCl_2_ (**B**). At time intervals indicated in the figure, aliquots of the solutions were treated with 3 mM phenylmethylsulphonyl fluoride (PMSF) to stop the digestion. The digests were analyzed by SDS-PAGE as described in Materials and Methods. (**A**) and (**B**), plots of disappearance of undigested actin; (C) SDS-PAGE patterns of non-modified (lanes a–d) and Cu^2+^-modified F-actins (lanes a’–d’) digested for 12 min are shown for Ca-F-actin polymerized with CaCl_2_ (lanes a, a’), Ca-F-actin polymerized with KCl (lanes b, b’), Mg-F-actin polymerized with KCl (lanes c, c’), and Mg-F-actin polymerized with MgCl_2_ (lanes d, d’). Apparent molecular masses of the digestion products are indicated.

The major subtilisin cleavage site in G-actin, at Met47 [44], becomes little accessible upon polymerization of actin [25,45]. It has previously been shown that Cu^2+^ binding to Ca-F-actin polymerized with 2 mM MgCl_2_ results in an exposure of this site [8]. We have compared the effects of Cu^2+^ on subtilisin digestion of Ca-F-actin and Mg-F-actin polymerized with either 0.1 M KCl, or with 2 mM CaCl_2_ or MgCl_2_, respectively (Figure 7). Time courses of decay of intact actin (Figure 7A, B) clearly show that the acceleration of F-actin digestion by Cu^2+^ binding depended on the type of both the polymerizing salt and tightly bound cation: the presence of KCl increased the resistance of F-actin subunits against proteolysis, and the presence of Mg^2+^ at the high-affinity site had an additional protective effect. 

**Figure 8:**
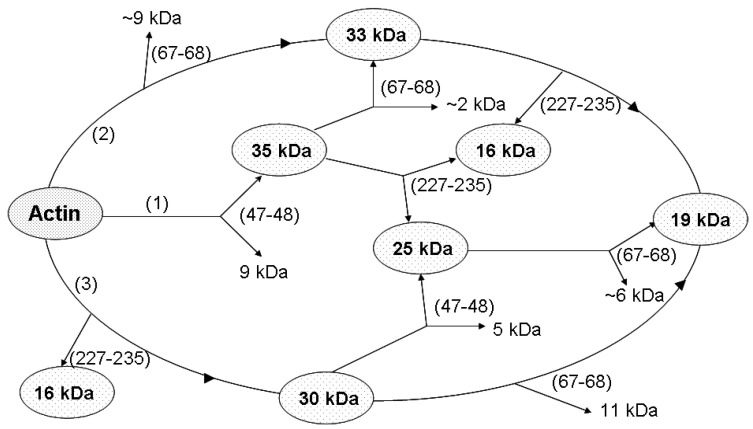
Scheme of G-actin proteolysis with susbtilisin. The numbers (1), (2), (3) represent three pathways of molecule degradation. Proteolysis products detected by SDS-electrophoresis are surrounded by ellipses. The polypeptide chain cleavage sites are given in parentheses.

**Figure 9:**
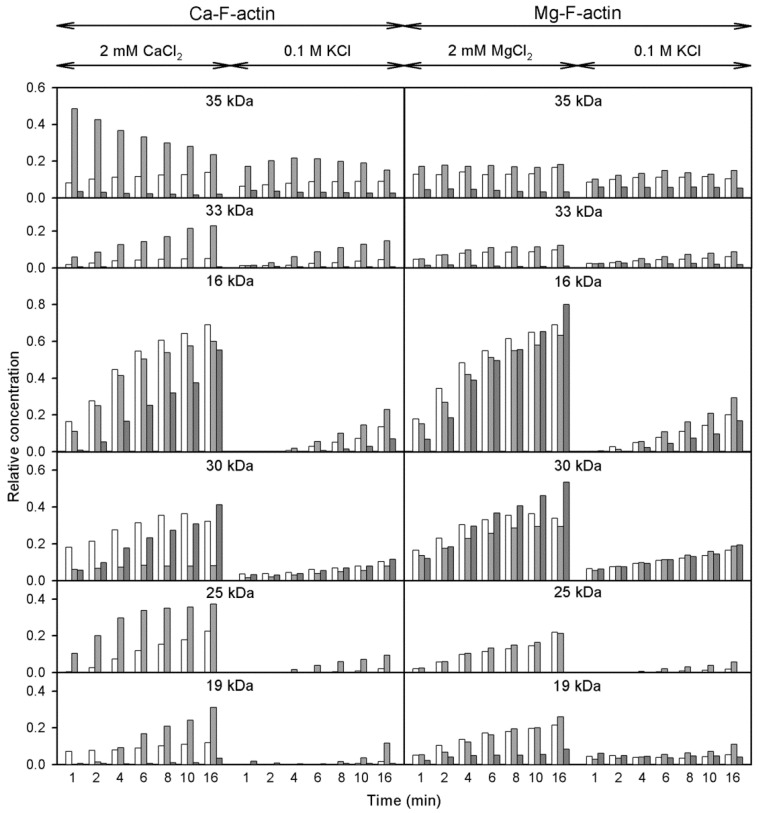
Analysis of the effects of solvent conditions on subtilisin digestion of unmodified and Cu^2+^-modified F-actin. Open, light grey bars and dark grey bars show control F-actin, Cu-modified F-actin, and phalloidin-modified F-actin, respectively. For more information see the text.

In addition to the cleavage at Met 47 within D-loop, that produces the 35-kDa C-terminal fragment (Figure 8, Pathway 1), subtilisin cleaves actin at two more sites, at Leu 67 in the nucleotide cleft (Figure 8, Pathway 2) [25], and within Segment 227-235 in SubDomain 4 (Figure 8, Pathway 3) [25,45] producing the C-terminal fragments of 33 and 16 kDa, respectively, and the corresponding N-terminal fragment of 30 kDa. Further fragmentation results in formation of the 25 and 19 products as well as additional accumulation of the 16 kDa fragment (Figure 8). Analysis of the rates at which these fragments are accumulated (Figure 9) allowed us to compare the effects of the tightly bound cation and polymerization conditions on accessibility of the cleavage sites.

Figure 9 (open bars) shows that in F-actin, in agreement with the F-actin models [3,4,5] and the earlier data [25,44], the bonds 47–48 and 67–68 are protected against proteolysis due to intersubunit interactions. In contrast, Segment 227-235 that is partially exposed [4,21] was cleaved intensively under all the conditions studied. Thereby only the bond 67–68 is somewhat more accessible in Mg-actin vs. Ca-actin, both in intact actin and in the 30 kDa-fragment. In F-actin polymerized with MgCl_2_ or CaCl_2_, binding of Cu^2+^ at Cys374 (Figure 9, light grey bars) resulted in enhancement of the cleavages at Met47 and Leu67, producing the 35 kDa and 33 kDa fragments, respectively (Figure 8, Pathways 1 and 2). However, the Cu^2+^ binding did not produce practically any effect on Segment 227-235. This is evidenced by the absence of a clear effect of Cu^2+^ on the accumulation of the C-terminal 16 kDa fragment, which is a direct product of proteolysis at this site, and the accumulation of the N-terminal 30 kDa fragment (Figure 8, Pathway 3). In Ca- but not in Mg-actin the strong cleavage of bond 47–48 was followed by further degradation of the 35 kDa fragment indicating destabilization of intersubunit contacts involving D-loop by Cu^2+^ and stabilization of this region by tightly bound Mg^2+^. In Mg-actin the area of Leu 67 was also protected.

In the presence of phalloidin (Figure 9, dark grey bars) the cleavage at 47–48 and 67–68 was strongly inhibited, consistently with the protection by phalloidin of the cleavage sites in the D-loop and in the interdomain cleft. At Segment 227-235, the cleavage was only partially inhibited in Ca-actin polymerized with CaCl_2_ and practically not affected in Mg-actin polymerized with MgCl_2_. 

When actin was polymerized with KCl, the cleavage at all the subtilisin-sensitive sites was inhibited both in the presence and absence of Cu^2+^, confirming stabilization by ionic strength of the intersubunit contacts [25]. It is also noteworthy, that in F-actin polymerized with KCl Cu^2+^-induced acceleration of the cleavage at Segment 227-235 was observed.

Thus, modification of actin C-terminus by Cu^2+^ binding increases the solvent accessibility of D-loop both in 2 mM CaCl_2_ or MgCl_2_ and in 0.1 M KCl, and phalloidin abolishes this effect. In contrast, the solvent accessibility of Segment 227-235 is affected by both Cu^2+^ binding and phalloidin only if 0.1 M KCl is present in the polymerizing solution.

### 2.5. Probing the Effect of KCl on Susceptibility of G-Actin to Limited Proteolysis with Subtilisin

**Figure 10:**
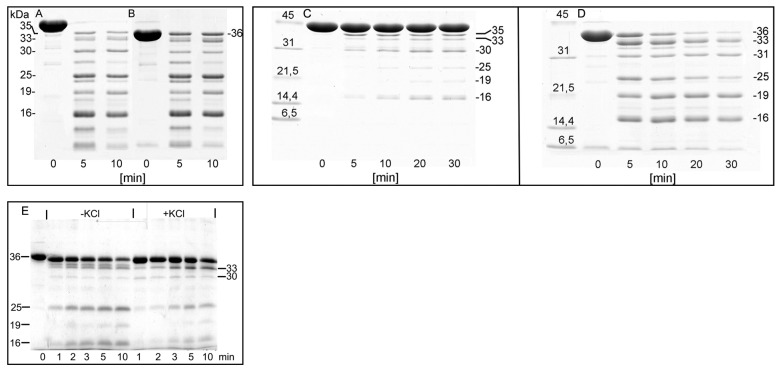
Effect of KCl on the susceptibility of Segment 227-235 of G- actin to proteolysis by subtilisin. Electrophoretic images with 24 µM native (**A**) and ECP-modified G-actin (**B**), before (time 0) and after 5 and 10 min of subtilisin digestion at 25 °C. Native (**C**) and ECP-modified (**D**) actin was polymerized with 0.1 M KCl. After polymerization, actin (at a concentration of 24 µM) was digested with subtilisin for 5, 10, 20 and 30 min at 25 °C. 10 µM ECP-cleaved Ca-G-actin (**E**).

At the early stage of actin polymerization, KCl could protect G-actin against proteolysis due to a putative activation of actin monomer at the pre-nucleation step of actin polymerization [46]. Therefore the KCl-dependent protection of F-actin prompted us to examine the effect of KCl on sensitivity of G-actin to proteolysis with subtilisin. To avoid the KCl-induced assembly of actin monomers we used the non-polymerizable Ca-G-actin cleaved between Gly42-Val43 with bacterial protease ECP32/grimelysin (ECP-actin) [47]. Treatment of both intact and the ECP-actin with subtilisin (Figure 10) produced the same cleavage pattern as was shown previously for G- and F-actin [45,46], with the fragments of 35, 33, 30, 25, 19 and 16 kDa formed (Figure 10A,B). 

The presence of 0.1 M KCl strongly inhibited the cleavage of intact actin with subtilisin (Figure 10C) while the ECP-actin was digested intensively (Figure 10D). In ECP-actin, 0.1 M KCl decreased the yield of all the subtilisin-produced fragments, including formation of the 25 and 16 kDa fragments, corresponding to the cleavage within Segment 227-235 (Figure 10E). Thus, the KCl-induced protection of this segment is due to conformational changes within actin subunit rather than to the effect of the intersubunit contacts. These structural changes may, however, be propagated along the subunit to tighten the intersubunit contact site. was digested with subtilisin at an enzyme/protein mass ratio of 1:50 for 1,2,3,5 and 10 min at 25 °C, before (-KCl) and after incubation for 10 min with 0.1M KCl (+KCl). The digestions were stopped with 3 mM PMSF. SDS-PAGE patterns of the digests are shown. The first lane, marked 0, shows ECP G-actin. Apparent molecular masses of the digestion products calculated on the basis of their electrophoretic mobility are indicated. 

## 3. Discussion

Analysis of the G- and F-actin structures available to date shows that in addition to D-loop conformationally flexible clusters include another part of Subdomain 2 such as Residues 61–65, Residues 195–205 and 232–246 in Subdomain 4, and both of the actin termini located in Subdomain 1, Residues 1–4 and 350–375 with the most terminal residues frequently disordered and absent in the crystal structures [48,49]. Several of these most variable and flexible regions represent specific insertions in actin sequence that play key role in maintaining the helical filament structure [50], and, according to the existing F-actin models [3,4,5,6] are involved in the actin-actin contacts in the polymer. Therefore, it is reasonable to assume that actin filament dynamics is coupled to the high conformational flexibility exhibited by the protein. 

It is well documented that conformation of G-actin flexible loops can be modulated by the kind of cation bound at high-affinity site. Investigations of Ca^2+^/Mg^2+^-dependent conformational transitions in ATP-G-actin using various techniques consistently show a change within the C-terminal segment or in its vicinity [51,52,53,54,55], in Segments 18–29 in Subdomain 1 [56], 202-204 in Subdomain 4 [55,57] and in Segments 61–69 in Subdomain 2 [55,56]. Proteolytic cleavage of ATP-G-actin at Gly42 and Met47 [54] and fluorescence of dansyl probes covalently attached to Gln41 [41] failed to detect significant cation-dependent changes. However, moderate differences in solvent accessibility of His40, Met44, and Met47 in MgATP- and CaATP-G-actin were observed using radiolytic footprinting [55]. The results of our work show that the presence of Mg^2+^ at the high-affinity cation binding site of Cu-modified actin polymerized with MgCl_2_ strongly enhances the filament subunit exchange and promotes the filament instability. These changes correlate with the increased accessibility of C-terminal segment, as can be judged from differences in the rates of Cu^2+^ binding, and with a putative rearrangement of Subdomain 2 resulting in protection in this actin of the subtilisin-sensitive cleavage sites at Met47 and Leu67. Such rearrangement is suggested by the model of Mg-actin based on crystallographic and radiolysis data [58]. The difference in the rates of the Cu^2+^-induced decrease in the solvent accessibility of DED-Mg-F-actin vs. DED-Ca-F-actin (this work) is also consistent with this model.

**Figure 11:**
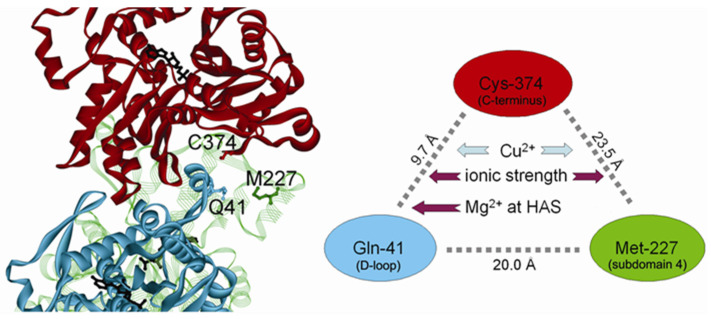
A model of the actin filament illustrating spatial relationships between the C-terminus, D-loop, and Segment 227-235 in Subdomain 4 of actin. The scheme on the right shows the distances between the α-carbons of Cys-374, Gln-41 and Met-227 located on 3 different monomers. The coordinates of the structure [2] were downloaded from K. Holmes Web site (ftp:\\149.217.48.3) and rendered with ViewerLite software (Accelrys Corp.). HAS denotes the high-affinity site for divalent cation binding.

Another factor affecting flexibility of the loops is ionic strength of polymerizing solution [20,25,59,60]. Consistently with the earlier data [35], in the presence of 0.1 M KCl, the filament subunit exchange was 2–3-fold lower than that in the MgCl_2_ or CaCl_2_-polymerized actin species. This effect correlates with the diminished accessibility of both D-loop and Segments 227-235 on the opposite filament strand (Figure 11).

**Figure 12:**
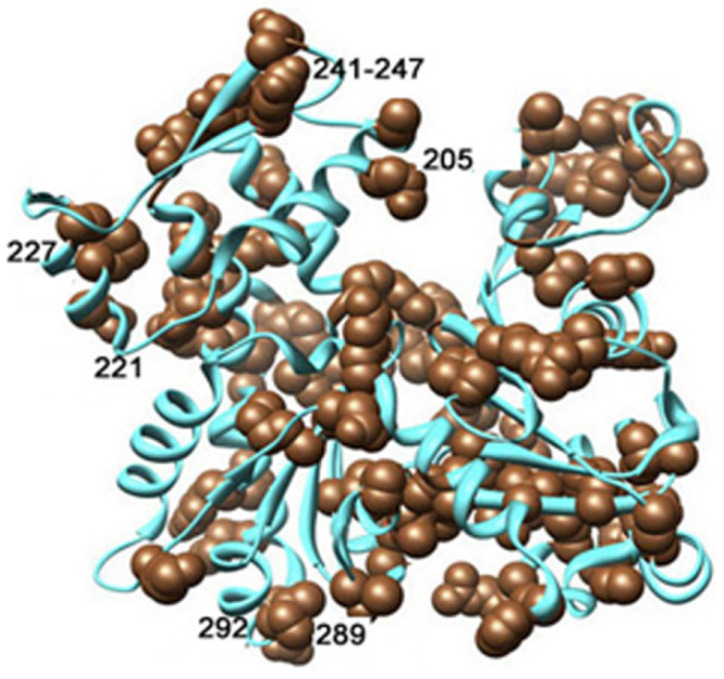
Nemaline myopathy actin mutations spread all over the 3D structure of actin molecule are shown; mutated residues are marked as brown spheres [61]. The numbers are added to show mutations of the amino acid residues discussed above. Reproduced from [61] with permissions from Elsevier Licence 4982111299506.

Even more striking effect of ionic strength on dynamic nature of intermonomer interface was revealed at the thorough analysis of the subtilisin cleavage dynamics. This analysis showed that an increased susceptibility of Segment 227-235 to subtilisin cleavage and abolishment of this effect by phalloidin took place only in KCl-polymerized actin. In actin polymerized with 2 mM CaCl_2_ or MgCl_2_ accessibility of Segment 227-235 did not depend on the presence of Cu^2+^ at the monomer C-terminus. This difference is consistent with an ionic-strength-dependent conformational change that modulates involvement of Segment 227-235 in stabilization of the intermonomer interface. KCl may restrict mobility of α-helix encompassing part of Segment 227-235 and/or be bound to Asp236 adjacent to Segment 227-235. This, in turn, can be transmitted to Loop 241-247 at the top of Subdomain 4 involved in the interface with Subdomain 3 of the subunit above [3,5,6]. This contact can also be stabilized by KCl binding at a discrete cation binding site comprising Residues 202-206 and 285-288, 290 of adjacent filament subunits [58]. It is known that mutations in Segment 227-235 and amino acid residues in its intermolecular contact interface are often associated with myopathies characterized by damage of the contractile apparatus and destabilization of the filaments [27,61,62]. These myopathy-associated mutations in Segment 227-235 and in the intermolecular interface of this segment are shown in Figure 12 [61].

In the cryoelectron microscopy images of F-actin [5,6], D-loop contacts with Residues 168–169, 144 and 352 along the filament, as well as with the hydrophobic plug across the filament, whereas C-terminus of actin monomer is not involved in the intermonomer contacts. 

However, allosteric effects of the modified Cys374 on Subdomain 2 [63,64,65], as well as the effects of the D-loop modification on the C-terminus [66,67] are documented. It is plausible, therefore, that the Cu^2+^-induced modifications of the C-terminus allosterically transmitted to D-loop modify D-loop contacts with Subdomain 3, thus destabilizing the filament. This is consistent with the Cu^2+^-associated increase in susceptibility of the sites at Met47 and Leu67 to subtilisin cleavage in F-actin (this work).

Taken together, the results of this work provide experimental evidence for involvement of Segment 227-235 of Subdomain 4 in the salt-induced stabilization of contacts within the actin filament. This stabilization is observed at ionic conditions close to physiological and weakens by the modification of the C-terminus as well as by myopathy-associated mutations within Segment 227-235. Such effects may be of physiological importance in view of the fact that a hydrophobic pocket in Subdomain 1 of actin, located at the entrance of the hydrophobic cleft between Subdomains 1 and 3 where the C-terminus is situated, is a target for numerous G-actin- and F-actin-binding proteins controlling actin dynamics in the cell [68,69]. Mutations of actin amino acid residues within a hydrophobic pocket in Subdomain 1 of actin can also be involved in these interactions [27,28,70,71]. 

## 4. Material and Methods

### 4.1. Reagents

EGTA (ethylene glycol-bis(β-amino ethylether)-N,N,N′,N′-tetraacetic acid) Tris (hydroxymethyl)aminomethane), Hepes (4-(2-hydroxyethyl)-1-piperazineethanesulfonic acid), phalloidin, subtilisin Carlsberg, sucrose (Sigma Ultra), dithiothreitol, and ATP were from Sigma Chemical Co. (St Louis, MO, USA). Dansyl ethylenediamine (DED) was purchased from Molecular Probes (Eugene, OR, USA). All other chemicals were of analytical grade. Microbial transglutaminase was a generous gift from Drs. M. Motoki and K. Seguro.

### 4.2. Protein Preparations

Rabbit skeletal muscle actin was prepared according to [72] with an additional purification by gel filtration on a Sephadex G-100 column. G-actin in buffer G (2 mM Hepes or Tris-HCl, pH 7.6, 0.2 mM ATP, 0.1 mM CaCl_2_, 0.2 mM dithiothreitol, 0.02% NaN_3_) was stored on ice and used within a week. Labeling of actin at Gln41 with DED via the transglutaminase reaction [73] was performed as described by [41]. Unbound dye and protein aggregates were removed by gel filtration on a Sephadex G-100 column using buffer G as an eluent. In all experiments on Cu-modified actin, buffer G containing Tris-HCl devoid of DTT was used. Actin labeled with N-(1-pyrenyl)iodoacetamide at Cys374 was prepared as described by Cooper et al. [74]. Mg-G-actin was prepared by a 5-min incubation of Ca-G-actin with 0.2 mM EGTA/0.1 mM MgCl_2_ at room temperature. In experiments on Cu-modified actin, EGTA was then removed from the solution by fast gel filtration on a Sephadex G-25 column (PD-10, Amersham Biosci., Uppsala, Sweden) equilibrated with 2 mM Tris-HCl, pH 7.6, 0.5 mM ATP, 0.05 mM MgCl_2_, 0.02% NaN_3_. The gel-filtered G-actin was immediately polymerized with 2 mM MgCl_2_ or 0.1 M KCl. Cu^2+^-modified F-actins were obtained by incubation of 10 µM F-actin solutions with 10 µM CuCl_2_ at room temperature for 15 min.

Protein concentration was determined spectrophotometrically using an absorption coefficient of 0.63 mL·mg^−1^·cm^−1^ at 290 nm for G-actin [75].

### 4.3. Measurements of Cu^2+^ Binding

The binding of Cu^2+^ to F-actin was monitored by recording changes in the absorption at 348 nm [76] or the changes in the fluorescence of DED-actin [8]. 

### 4.4. Steady-State ATPase Activity Measurements

The rates of ATP hydrolysis in steady-state F-actin solutions were measured in the presence of 0.5 mM ATP, at 25 °C. In aliquots of the solution withdrawn after various time intervals the reaction was quenched by addition of an equal volume of 0.6 M ice-cold perchloric acid, precipitated protein was removed by centrifugation, and released P_i_ was determined by the Malachite Green method [77]. 

### 4.5. Determination of the Relative Number Concentration of Actin Filaments

The relative number concentration of the filaments in F-actin solutions was determined using a slightly modified procedure described by [78]. Aliquots of 10 µM F-actin solutions were diluted 10-fold into 3 µM 10% pyrenyl-labeled Ca-G-actin supplemented with 0.1 M KCl several seconds before addition of F-actin. The solutions were gently mixed in fluorescence cuvettes by inversion 5 times (this resulted in a dead time of about 10 s), and the increase in pyrenyl fluorescence intensity associated with polymerization of the labeled G-actin on non-labeled F-actin was recorded at 25 °C. The relative number concentration of filaments in F-actin solutions used as polymerization seeds is given by the ratio of filament elongation rates derived from the slopes of the initial linear parts of the fluorescence curves.

### 4.6. Critical Concentration for Actin Polymerization

Critical concentrations for polymerization of unmodified and Cu^2+^-modified actins were measured by determination of the steady-state polymer concentration as a function of actin concentration. Samples of F-actin solutions were diluted into the polymerization buffer (supplemented with CuCl_2_ in case of the modified actin) to various final concentrations of actin. After equilibration at 25 °C for 2 h, light scattering intensity of the solutions was measured at 450 nm. Critical concentrations were obtained by linear extrapolation to zero of the plots of light scattering intensity as a function of actin concentration. 

### 4.7. Proteolytic Digestions

G-actin and F-actin (10 µM) were digested with subtilisin at 25 °C at an enzyme/protein mass ratio of 1:50 and 1:200, respectively. The digestion was terminated by addition of 3 mM phenylmethylsulphonyl fluoride (PMSF), and the digests were analyzed by SDS-PAGE as described earlier [19]. Coomassie Blue-stained gels were scanned in an AGFA Arcus 1200 scanner. The protein bands were quantified using the Molecular Dynamics Image Quant version 3.3 software.

### 4.8. Fluorescence Anisotropy Measurements

Steady-state fluorescence anisotropy measurements were collected in a Fluorolog-3 spectrofluorometer (Spex Industries, Edison, NJ, USA) using motor-driven Glan-Thompson polarizers. The excitation and emission wavelengths were 334 and 535 nm, respectively. The anisotropy data were corrected for background and unequal sensitivity of the detection system for horizontally and vertically polarized light. All corrections and the computation of anisotropy values were done with the manufacturer-provided software. The anisotropy data obtained at different concentrations of sucrose (0–46% *w*/*v*) were analyzed using the Perrin equation [79]:

(1) $$\frac{1}{A}=\frac{1}{A_{0}}+\frac{\tau RT}{A_{0}V_{a}\eta }$$

where *A* is the measured anisotropy, *A*_0_ is the fundamental anisotropy of the chromophore, *τ* is fluorescence lifetime, *R* is the gas constant, *V_a_* is the molecular volume of the rotating unit, *T* denotes absolute temperature, and *η*, viscosity of the solution. If *τ* and *V_a_* are constant the plot 1*/A* vs. *T/η* is linear and the y-intercept corresponds to the anisotropy at infinite viscosity of the solution and should equal to 1*/A*_0_. If segmental motion takes place, *A*_0_*^app^* is less than *A*_0_ and the angle of rotation *θ* can be calculated from the equation:

(2) $$\frac{A_{0}^{app}}{A_{0}}=\frac{3{cos}^{2}\theta -1}{2}$$

### 4.9. Fluorescence and Light Scattering Measurements

The measurements were carried out in a Spex Fluorolog-2 spectrofluorometer (Spex Industries, Edison, NJ, USA). The fluorescence intensity of pyrenyl-labeled actin was recorded at 407 nm after excitation at 365 nm [80]. Excitation and emission wavelength for the fluorescence of DED-labeled F-actin were set, respectively, at 334 nm and 525 nm. Intensity of light scattered at 90° was measured at 450 nm. 

### 4.10. Electron Microscopy

F-actin solutions were applied to carbon-coated copper grids without dilution. The filaments were negatively stained with 1% (*w*/*v*) uranyl acetate after washing the grid with a few drops of the same uranyl acetate solution to remove an excess material. Specimens were examined in a Joel JEM 1200 EX electron microscope at an accelerating voltage of 80 kV.

## Figures and Tables

**Table 1:** Rates of the steady-state ATP hydrolysis in solutions of Cu^2+^-treated F-actins.

ATP Hydrolysis (mol/mol actin/h)				
Actin Species	Polymerizing Salt	N	Measured	Normalized to the Same N
Ca-F-actin	0.1 M KCl	1	0.66	0.66
Ca-F-actin	2 mM CaCl_2_	2.4	0.60	0.25
Mg-F-actin	0.1 M KCl	2.2	0.61	0.28
Mg-F-actin	2 mM MgCl_2_	7.4	5.73	0.77

Experimental values for the steady-state ATP hydrolysis are from the data in Figure 1A. N denotes relative number concentration of filaments in the F-actin solutions calculated from the data in Figure 2 with the value for Ca-F-actin polymerized with 0.1 M KCl taken as 1.

## Data Availability

Data available on request due to restrictions eg privacy or ethical.
